# Towards a comparative science of emotion: Affect and consciousness in humans and animals

**DOI:** 10.1016/j.neubiorev.2019.11.014

**Published:** 2020-01

**Authors:** Elizabeth S. Paul, Shlomi Sher, Marco Tamietto, Piotr Winkielman, Michael T. Mendl

**Affiliations:** a Bristol Veterinary School, University of Bristol, Langford House, Langford, Bristol, BS40 5DU, UK; b Department of Psychology, Pomona College, Claremont, CA, USA; c Department of Medical and Clinical Psychology, Tilburg University, Tilburg, the Netherlands; d Department of Psychology, University of Torino, Torino, Italy; e Department of Psychology, University of California, San Diego, La Jolla, CA, 92093, USA; f Faculty of Psychology, SWPS University of Social Sciences and Humanities, 03-815, Warsaw, Poland

**Keywords:** Affect, Animals, Componential, Consciousness, Interoception, Neural correlates, Subjective emotion, Unconscious emotion

## Abstract

The componential view of human emotion recognises that affective states comprise conscious, behavioural, physiological, neural and cognitive elements. Although many animals display bodily and behavioural changes consistent with the occurrence of affective states similar to those seen in humans, the question of whether and in which species these are accompanied by conscious experiences remains controversial. Finding scientifically valid methods for investigating markers for the subjective component of affect in both humans and animals is central to developing a comparative understanding of the processes and mechanisms of affect and its evolution and distribution across taxonomic groups, to our understanding of animal welfare, and to the development of animal models of affective disorders. Here, contemporary evidence indicating potential markers of conscious processing in animals is reviewed, with a view to extending this search to include markers of conscious affective processing. We do this by combining animal-focused approaches with investigations of the components of conscious and non-conscious emotional processing in humans, and neuropsychological research into the structure and functions of conscious emotions.

## Highlights

• Emotions comprise conscious, behavioural, physiological and cognitive elements.
• Neural correlates of conscious emotion can be investigated in humans and animals.
• Contemporary theories of consciousness have differing implications for animals.

## 1 Introduction

For humans, emotions are quintessentially about feelings: consciously experienced, subjectively focused, reportable, affective states. Measuring these states seems easy enough; all we need to do is ask. On closer inspection, complications arise: Different methodologies and questionnaires may tap different facets of felt emotion or affect, and there may not always be perfect correspondence between their findings. Some people will be poor at recognising or articulating their emotions, while others will lie about how they actually feel. And when emotion reports have a retrospective component, they can be subject to constructive biases of memory (Redelmeier and Kahneman, 1996). But, ultimately, the gold standard of subjective emotion measurement in healthy adult humans remains linguistic report (Barrett, 2006a; Bradley and Lang, 2000; LeDoux, 2014a, LeDoux and Hoffmann, 2018; Oatley and Jenkins, 1996). Unfortunately, this state of affairs leaves a major problem for anyone interested in conscious emotional or affective states in non-human animals, because animals cannot tell us how they feel. In fact, the problem is twofold. First, we do not know for sure which species have the capacity for consciousness of any kind, emotional or other (e.g. Dawkins, 2000, 2001, 2017; LeDoux, 1996; Macphail, 1998; Rolls, 1999, 2005, 2007; although see Panksepp, 1994, 2005; and Wemelsfelder, 2001, for alternative views). And second, for those species with a capacity for consciousness, we do not have methods for establishing whether and what sorts of conscious emotions they experience.

These problems are relevant to almost all scientists studying non-human affect. The expanding field of affective neuroscience relies heavily on comparative studies of emotion or emotion-like states in both humans and animals (e.g. Alcaro et al., 2007; Berridge, 2000; Buchel and Dolan, 2000; Lang et al., 2000; LeDoux, 2000; Phelps and LeDoux, 2005). Much animal-based research in psychopharmacology makes use of parallels between the emotional systems of humans and other species (e.g. Frazer and Morilak, 2005; Haug and Whalen, 1999; Pawlak et al., 2008). But we cannot make confident comparisons between humans and animals in the critical domain of conscious affect. For example, when pharmacological interventions have parallel effects on aspects of human and animal behaviour, it is reasonable to suppose, but difficult to decisively demonstrate, that they have similar effects on feelings or conscious experiences (e.g. Kregiel et al., 2016; Vera-Chang et al., 2018). Animal welfare researchers face a similar problem. The evaluation of interventions to improve the well-being of animals is limited by our capacity to identify the character, or even the existence, of their conscious affective states (Dawkins, 2017; Mendl and Paul, 2004).

Traditionally, the topic of conscious affect in animals has been regarded as all but taboo, with the possibility of conscious processes in animals having long been seen as fundamentally inaccessible to empirical investigation (e.g. LeDoux, 1996; Skinner, 1953). But in recent years there has been a rapid expansion of the fields of comparative emotion and affective neuroscience (e.g. see Anderson and Adolphs, 2014; de Waal, 2011; Mendl et al., 2010; Paul et al., 2005), and over a similar period, there has been an equally dramatic rise in research into the neuroscience of consciousness (e.g., Dehaene, 2014; Koch et al., 2016). Together and in turn, these developments have prompted some early signs of serious academic interest in the study of conscious affect and its evolutionary antecedents, with a number of recently published papers considering this difficult issue directly (Berridge, 2018; [Bibr bib1145][Bibr bib1520]; Smith and Lane, 2015, 2016). In the present review, we focus on the methodological and theoretical issues emerging from studies of human consciousness and human emotion which are directly pertinent to investigations of conscious affective processes in animals.

In Section 2, we start with a brief discussion of emotion terminologies in the context of humans and non-human animals (hereafter animals). In Section 3, we highlight the utility of the componential view of emotions – as multifaceted states comprising behavioural, physiological, cognitive and conscious components – in advancing animal emotion research in general, and research into conscious emotion in particular (e.g. Bradley and Lang, 2000; Clore and Ortony, 2000; Lang, 1993; LeDoux, 1996; Scherer, 1984). Section 4 sets the scene for developing comparative investigations of conscious emotion by briefly outlining the neural and information processing correlates of human consciousness, and discussing how these may be translated to animals. In Section 5, we narrow our focus to conscious emotion and review research and theory regarding its structure, highlighting possible similarities and differences between affective conscious experiences in humans and non-human animals. In Sections 6 and 7 we consider potential markers for conscious emotion, focusing on the search for its neural and cognitive correlates, and considering the ways in which this search could be applied.

Our aim is not to offer premature answers to questions about the distribution and character of nonhuman emotional experience. Instead, our goal is to gauge our current state of understanding of affect in humans and animals, to clarify the types of information that are needed to develop a new, comparative science of conscious emotion (Berridge, 2018; LeDoux and Hofmann, 2018; Paul and Mendl, 2018), and to illustrate the principles and potential pitfalls of this endeavour.

## 2 Terminology: emotion, affect, and feelings in humans and animals

Although extensively studied in humans, a universal definition of emotions is still contentious. In the 1980s, Fehr and Russell (1984, p. 464) wrote that “Everyone knows what an emotion is, until asked to give a definition. Then, it seems, no one knows.” Kleinginna and Kleinginna (1981) considered 92 definitions and 9 sceptical descriptions produced by scientists in the field, illustrating the lack of consensus that underlies the concept of emotion and its usefulness in the scientific framework. Having said this, most human researchers accept some version of the componential view of emotion (as elaborated in Section 3), in which it is defined as a state characterized by *loosely* coordinated changes in the following five components: (i) feeling—–changes in subjective experience, (ii) cognition—–changes in attentional, perceptual, and inferential processes (appraisals), (iii) action—–changes in the predisposition for or execution of specific responses, (iv) expression—–changes in facial, vocal, postural appearance, and (v) physiology—–changes in physiological and neural activity.

The use of terms and definitions in the context of animal emotions has long been a matter of confusion and controversy (e.g. see Duffy, 1934; Mandler, 1975). Whether words such as “emotion”, “fear”, “sadness” or “joy” should ever be used when talking about animals has been extensively debated (e.g. see Berridge, 2018; Damasio, 1999; Davidson, 2003; LeDoux, 1996, 2014a; LeDoux and Hofmann, 2018; Winkielman and Berridge, 2004). LeDoux (1996, 2012, 2014a, LeDoux and Brown, 2017; LeDoux and Hofmann, 2018) has suggested that the words “emotion” and “fear” by themselves imply human-like conscious or phenomenal states and inner experiences, and proposes instead that terms such as “emotional processing” and “survival circuits” should be used with regard to animals. Damasio (1994) used the phrase “primary emotions” to refer to the automatic emotional processes in animals and humans that are not necessarily conscious, and “feelings” to refer to those that are. Berridge and colleagues (Berridge, 2000; Berridge et al., 2009; Winkielman and Berridge, 2004) have used quotation marks around the terms “liking” and “wanting” to highlight agnosticism regarding the presence or absence of conscious experience of these states in experimental animals.

We take the view that, in the absence of an entirely new nomenclature, “emotion” in its broadest sense, remains convenient to use as an umbrella term when referring to the whole variety of observed aspects of emotional or emotion-like processing in a range of species, whether consciously experienced or not (e.g. see also Berridge, 2018; Berridge and Winkielman, 2003; Phelps and LeDoux, 2005; de Waal, 2011). Thus, we are happy to use “animal emotion” to refer to this area of study. But many authors have opted for expressions such as “emotion-like”, “anxiety-like” and “fear-like” to refer to specific states in animals that bear behavioural and/or physiological resemblance to human emotions, yet may or may not be conscious (e.g. Perry et al., 2016), and we also adopt this convention here.

We also note that the terms “affect” and “affective state” are used widely in the animal literature. In human psychology, the word “affect” often refers to felt states that are *valenced* (consciously experienced as pleasant or unpleasant, positive or negative). These include emotions and the valenced components of sensations such as pain (i.e. the affective as opposed to sensory component of pain; Hofbauer et al., 2001). Accordingly, we similarly use “affect” to refer to valenced states in animals (often associated with approach/withdrawal, or reward/punishment), without implying that such states are necessarily consciously experienced. Note that emotions, with their quick onset, shorter duration, and a specific target, can also be distinguished from slow-onset, long-duration, diffused affective states like moods. As such, the term “affect” is useful in that it underpins both constructs, in humans and animals alike (e.g. Frijda, 2009; Mendl et al., 2010; Scherer, 2005a,b).

Finally, in human research, the terms “conscious emotion” and “subjective emotion” tend to be used synonymously, although “subjective emotion” is probably the more popular usage (e.g. Loewenstein et al., 2001; Pham, 1998; Schwarz and Clore, 1988). A restrictive definition of a subjective emotion is one in which an individual is not only experiencing an emotion consciously, but is also aware that they are the subject of that feeling – “I am happy”; “I feel depressed”. That is, it combines a notion of self-awareness with one of conscious experience. The idea of a conscious emotion, on the other hand, is somewhat looser, potentially indicating nothing more than a raw feeling without any accompanying sense of selfhood. In the present paper, therefore, we use the terms “conscious affect”, “conscious emotion”, and “feeling” when considering the possibility of consciously experienced emotional feeling occurring in animals, to avoid the implication that such a state necessarily involves conscious self-awareness.

## 3 The componential structure of emotion: conscious feelings as one component of emotional states

Emotions can be seen as complex, multifaceted events or processes incorporating a range of components that are expressed in a variety of ways: Consciously (or verbally – Bradley and Lang, 2000), neurally, physiologically, behaviourally, cognitively, and expressively (e.g. Bradley and Lang, 2000; Clore and Ortony, 2000; Frijda, 1986; Lang, 1993; Panksepp, 2003; Smith and Lane, 2015). Within this componential view (Scherer, 1984), the reportable, conscious component of emotion is the one that is often regarded as its central, even defining feature, starting from William James (James, 1884; Lieberman, 2019; Robinson and Clore, 2002). But it is also just one of many measurable facets (Berridge and Winkielman, 2003; Davidson, 2003). For example, in humans, emotions such as fear or anxiety involve changes in heart rate, heightened sympathetic nervous system activation, alterations of attention (e.g. toward threatening stimuli), subjective feelings of terror or dread, changes in voice and posture and increased behavioural tendencies to freeze or run away (e.g. Lang et al., 2000; LeDoux, 2003).

These facets can also be thought of in *functional,* as well as in measurement terms. They often act concurrently, collectively, and coherently within specific emotional episodes. But they can also operate independently, or partially independently, guiding types or modalities of response to emotion-eliciting stimuli. For example, the specific function of the elevated heart and respiration rate that occur during an episode of “fear” is to increase the supply of oxygenated blood to the peripheral musculature for the purposes of running away (Gray, 1994). Likewise, the specific function of opening the eyes wide in a fearful facial expression may be to enhance peripheral visual perception for the purposes of identifying and locating the source of threat (Susskind et al., 2008), with secondary functions related to social communication (Lee et al., 2013). Together, the overarching function of the coordinated changes in the components of the emotion labelled “fear” enables an individual to successfully avoid or escape from threat. So, the componential view of emotion encapsulates the idea that not only can the different facets of any one emotional response be measured in many different ways, but that they can also have a number of different functions, or sub-functions, within the wider emotional event (e.g. Blair, 2003).

The componential view of emotion allows investigation of animal emotions by measuring behavioural, physiological, neural and cognitive components without the need to first decide whether or not a conscious component exists in the species concerned. This has paved the way for rapid expansion of research into affective processes in animals (for reviews see: Anderson and Adolphs, 2014; Berridge, 2003; Bliss-Moreau, 2017; deWaal, 2011; Gygax, 2017; LeDoux and Hoffman, 2018; Mendl et al., 2010; Paul et al., 2005; Paul and Mendl, 2018; Panksepp, 2005). By thinking of the conscious component of emotions as separable, at least in part, from other components, we are also able to approach the study of conscious emotions from a functional perspective: what is it that an emotion does and, in particular, which of these functions may require a conscious component in particular? This opens up the possibility of investigating not only the neural markers or correlates of conscious emotional states, but also their functional correlates (i.e. their consequences or outcomes for the individual; e.g. see Tamietto and de Gelder, 2010; Celeghin et al., 2017; Diano et al., 2017; Mitchell and Greening, 2012).

In the next section we review literature pertaining to the neural correlates of general capacity for conscious experience in humans (NCCs) and consider the implications of existing (and competing) theories of conscious processing for our understanding of animal consciousness.

## 4 Neural correlates of consciousness

Research regarding the neural underpinnings of consciousness in humans has proliferated in recent decades, with significant developments occurring in studies of both the correlates of the *contents* of consciousness (e.g. correlates of conscious *vs.* non-conscious visual word processing; Dehaene et al., 2001) and correlates of *full* or *state* consciousness (e.g. correlates of dreaming vs dreamless sleep, or of conscious emergence from a vegetative state; Koch et al., 2016; Rosanova et al., 2012; Siclari et al., 2017). This rapidly expanding field has identified a range of potential markers of consciousness which may prove to be important in future comparative explorations of conscious experience in animals. A number of recent reviews consider the evidence for and against capacities for consciousness (of any kind) in a range of non-human species (e.g. Boly et al., 2013; Edelman et al., 2005; Edelman and Seth, 2009; Seth et al., 2005; Fabbro et al., 2015), and we do not canvass that evidence in detail here. Instead, we provide a brief overview of current findings with a view to using these to develop investigations of neural and functional correlates of conscious *emotion* across species.

But we begin with a note of caution. While the neuroscience of consciousness has garnered growing interest, it has also confronted serious methodological problems which have not been fully resolved. Typical studies employ some variant of the “contrastive method,” (Baars, 1997) in which neural activity is compared during conscious vs. non-conscious processing of similar stimuli. This method rests on two fundamental assumptions. First, the researcher must be able to reliably distinguish conscious vs. non-conscious forms of processing. However, even when simple stimuli are presented to healthy human adults, the identification or exclusion of consciousness can be far from trivial (e.g., in characterizing the nature of consciousness beyond the focus of attention; Cohen et al., 2016; Schwitzgebel, 2007), in turn generating disputes about how to apply the contrastive method (e.g., Block, 2011; Lamme, 2006; Dehaene et al., 2006). Second, if the presence or absence of consciousness can be identified, the researcher must be able to distinguish brain activity directly associated with consciousness from activity associated with prior processing which gates access to consciousness (“prerequisites” or “enabling conditions”) as well as from post-processing reflecting response preparation and other secondary effects of conscious information (“consequences”; DeGraaf et al., 2012; cf. Aru et al., 2012). While novel paradigms have recently been developed with the aim of separating conscious experience from its prerequisites and/or consequences (e.g., Boly et al., 2017; Pitts et al., 2014; Tsuchiya et al., 2015), controversy remains about the adequacy of these methods (e.g., Overgaard and Fazekas, 2016). In light of these core methodological challenges, it is prudent to exercise caution in drawing far-reaching conclusions from current findings; this is especially important to bear in mind when applications to animal welfare are considered (cf. Dawkins, 2015). Nonetheless, as research in this area advances, it is instructive to ask what candidate neuroscientific models of consciousness would imply, and what specific questions they would raise, about animal consciousness.

### 4.1 Neural substrates and information processing functions associated with consciousness

The modern search for neural correlates of consciousness (NCCs) in humans commenced about three decades ago (Crick and Koch, 1990; Marcel and Bisiach, 1988). Since then, a number of candidate NCCs have been proposed. NCC theories often identify (1) an information processing function, implemented in (2) a specific neural substrate, as the “minimally sufficient” condition (Koch, 2004) for conscious experience. The distinction between these two components can generate ambiguity in extending theories of human NCCs to animals, particularly for evolutionarily distant (i.e., non-mammalian) species (e.g. see Barron and Klein, 2016). For such species, it is possible that analogous functions may be implemented in non-homologous structures. An additional issue concerns the relationship between anatomical and functional homologies across mammalian and other more closely related species, in which anatomically similar structures may actually implement quite different cognitive functions. In light of these issues, the application of evidence from humans to the interpretation of animals can be far from straightforward. In particular, the implications of a particular NCC proposal for animal consciousness may depend critically on whether one considers the general information processing function, or the specific neural substrate (identified in human studies), to be essential for conscious processing in other species.

As an historical example, consider Descartes’ (1649) theory of the neurofunctional correlates of consciousness. At the functional level, Descartes argued that unbounded human capacities for language use and flexible generalization fall uniquely within the province of a conscious rational soul; at the neural level, he associated the unitary integrated character of conscious representations with the unpaired central structure of the pineal gland. Notwithstanding the presence of the relevant neural *substrate* (the pineal gland) in many animals, Descartes denied that they are conscious on account of the supposed absence of the relevant information processing *function* (unbounded generalization capacities). While Descartes’ account of the neural substrate of consciousness has of course long been eclipsed, current theories of the NCC raise similar questions of structural homology and functional analogy.

#### 4.1.1 Higher order theories of consciousness

Descartes’ scepticism about animal consciousness is echoed by some (though not all) modern proponents of higher-order theories of consciousness. At the functional level, these theories posit that a first-order representation is conscious only if it is also the object of an appropriate higher-order representation; different variants of the theory (e.g., higher-order thought [HOT] vs. higher-order experience [HOE] theories) differ in the properties they attribute to the relevant higher-order representations. While these theories have often been advocated on philosophical grounds, some defences (e.g., Lau and Rosenthal, 2011) appeal to evidence from neuroscience. These point to correlations between conscious awareness and activity in the dorsolateral prefrontal cortex (dlPFC), under conditions where task performance is matched for conscious and nonconscious stimuli (e.g., Lau and Passingham, 2006). This dlPFC activity is assumed to be the neural substrate of late processes of sensory metacognition involved in higher-order representation. However, the consistency and interpretation of the association between dlPFC activity and consciousness is a matter of continuing controversy (for contrasting perspectives, see Boly et al., 2017; Odegaard et al., 2017).

Critics (e.g., Dretske, 1995) have often objected to higher-order theories on the grounds that they would implausibly deny phenomenal consciousness to many, perhaps all, animals. In defending his version of HOT theory, Carruthers (1989,^1998^,^2000^,^2005^) has embraced this conclusion, contending that phenomenal consciousness is associated with specialized higher-order cognitive functions, is less integral to much ordinary human behaviour than we think it is, and is likely absent in most animals (see also Macphail, 1998). Other proponents, however, have argued that the relevant higher-order representations need not be as sophisticated as Carruthers and others suppose, leaving the door open to simpler forms of higher-order representation that may be sufficient for animal consciousness (Gennaro, 2004; Lau and Rosenthal, 2011). For higher-order theories of *emotional* consciousness specifically, see Rolls (1999, 2004) and LeDoux and Brown (2017). For other neurofunctional accounts that link consciousness with self-representation, as well as the representation of other minds, see Humphrey (1978) and Graziano (2013).

#### 4.1.2 Global workspace theory

In contrast with the higher-order theorist’s singular focus on self-representation, other NCC theories emphasize more general functions of information integration, flexible response selection, and coherent behavioural coordination. But these theories differ in the specific forms of integration and coordination they highlight and in the specific neural substrates they posit. The Global Workspace (GW) Theory, originally proposed by Baars (1988), equates consciousness with the broadcasting of selected information across a network of modular processors. In a GW architecture, isolated modules operate automatically, unconsciously, and in parallel; collectively they have high information capacity, but, working in isolation, are only able to perform routine functions appropriate to familiar situations. When novel situations arise for which isolated modules are unprepared, a subset of information is selected for entry into the GW, where it is broadcast across the entire network of specialist modules. Information sharing in the GW enables processing that is integrated, coordinated, and flexible, but the narrow information bandwidth of the GW entails accompanying costs in speed and efficiency. Dehaene and Naccache (2001) proposed that the neural substrate of the functional GW architecture in humans consists of association areas, principally in frontal and parietal cortex, whose widespread connections give them a central role in GW selection and broadcasting.

This neuronal GW theory predicts that, in experimental contrasts between conscious and matched unconscious processing, similar activity should be elicited in relevant local modules as part of a fast, initial forward-pass of processing. But conscious processing should be uniquely associated with a late “global ignition” of a distributed pattern of reverberating activity, with special involvement of fronto-parietal association areas. This core prediction finds support in a diverse range of clinical and experimental comparisons, including masked vs. visible stimuli (Dehaene et al., 2001), processing within vs. outside the attentional blink (Sergent et al., 2005), behaviour in sleepwalking vs. wakefulness (Bassetti et al., 2000), and in sensory responses (Laureys et al., 2002) as well as behavioural output (Plum et al., 1998) in vegetative state patients vs. healthy subjects. More recently, however, some researchers have argued the late global activation seen in these studies may reflect the post-processing *consequences* of consciousness, particularly those associated with stimulus report. This interpretation is bolstered by recent findings from “no-report” and related paradigms, which limit post-processing demands and which have yielded evidence for earlier, more posterior, and less widely distributed correlates of visual consciousness (Pitts et al., 2014; Tsuchiya et al., 2015; Koch et al., 2016).

In assessing implications for animal consciousness, it is useful to distinguish between two possible variants of GW theory. A broad version of the theory would ascribe consciousness to any functionally similar GW architecture, however physically realized. A narrow GW theory, in contrast, would specifically associate consciousness with the proposed neural substrate of the GW in humans (distributed neocortical modules linked by fronto-parietal association areas). Both versions of the theory would provide support for some non-human consciousness. For example, “global ignition” events with strong PFC activation are observed when rhesus monkeys detect (as indicated by a saccade to the target) a visual image flashed close to the sub/supraliminal threshold, but not when they fail to detect the same image on other trials (van Vugt et al., 2018). But broad and narrow GW theories may carry different implications for evolutionarily remote (e.g., non-mammalian) species. For example, birds confront their own problems of perceptual integration and behavioural coordination, and it is not unreasonable to ask whether they might also be solved by a GW architecture of some kind. And certain avian families such as the corvids (crows, ravens, jays, etc.) demonstrate cognitive skills that would be expected to require high-level cognitive processing in a mammal (Güntürkün and Bugnyar, 2016). However, on the (speculative) assumption that bird brains indeed employ a GW architecture (suggesting consciousness, on the broad view), its neural substrate would differ, more or less radically, from ours (calling consciousness into doubt, on the narrow view). When a corvid makes use of a novel tool (Bird and Emery, 2009) or appears to recognize itself in a mirror (Prior et al., 2008), is there global ignition of the avian pallium? A number of commentators have speculated about such processes in birds and other non-mammalian vertebrate species (e.g. Braithwaite, 2010; Butler et al., 2005; Cabanac et al., 2009; Paradis and Cabanac, 2004; Seth et al., 2005). Still farther afield, Tye (2017, pp. 153–156) argues that bee brains may have a miniature global workspace architecture of their own.

#### 4.1.3 Integrated information theory

Integrated Information Theory (IIT; Tononi, 2008; Tononi et al., 2016) shares GW theory’s broad emphasis on integration, but does not tie this general function to a postulated GW or any other specific mechanism. Instead, IIT proposes a formal measure Φ of the quantity of “integrated information” in any physical system whatsoever – that is, of information encoded in the whole system that is lost whenever the system is divided into parts. Unlike most NCC theories, in which consciousness is an all-or-none dichotomy (Sergent and Dehaene, 2004), IIT treats consciousness as a continuous variable, with higher Φ values indicating more (i.e. fuller) consciousness. The claim is that neural activity in structures like the cerebellum, with its independent modular construction (resulting in low Φ), are largely unconscious, while activity in the thalamocortical complex, with its extensive differentiation (information) and interaction (integration), is high in Φ, and hence also in consciousness. From an IIT vantage point, the NCC is likely to have a broad thalamocortical distribution, with a finer delineation of its boundaries requiring a closer study of the brain’s effective connectivity (i.e., how causal perturbations propagate across, and come to be represented in, the entire network). Empirical evidence for IIT comes from observed correlations between the apparent emergence of consciousness and rises in effective connectivity, in different sleep stages (Massimini et al., 2005), anaesthesia (Ferrarelli et al., 2010), and neurological disorders (Casali et al., 2013; Casarotto et al., 2016; Rosanova et al., 2012). Because IIT does not posit a specific mechanism of integration, it (unlike GW theory) is not threatened by recent evidence suggesting a more posterior NCC for perception (Koch et al., 2016). However, IIT faces challenges of its own. First, as has often been noted, the exact computation of Φ is intractable for even moderately complex systems, limiting the precision with which quantitative predictions of the theory can be tested. Second, it has recently been argued that IIT entails improbable assignments of superhuman consciousness to certain trivial computational systems (see Aaronson, 2014, and the ensuing online debate).

An IIT perspective would open up a broad and remarkably open-ended view of the possible distribution of animal consciousness. For IIT does not tie consciousness to any specific functional mechanism *or* physical substrate; any functional system with sufficiently high Φ will possess it. Therefore, while the presence of structures homologous to those with high Φ in humans is strong evidence for consciousness, the absence of such structures is not strong evidence against it. Distant evolutionary relatives may achieve comparable information integration in divergent ways, potentially leading to a diverse array of quite different NCCs across the animal kingdom. But we needn’t stop with animal consciousness; it has been noted that IIT suggests something like a graded panpsychism, in which consciousness may be distributed in varying degrees across inanimate as well as animate systems in the physical world (Tononi and Koch, 2015).

#### 4.1.4 The role of the brainstem

While the theories summarized above all localize the human NCC in the cerebral hemispheres, others have suggested a primary neural substrate for conscious experience in the upper brainstem. This suggestion, which goes back to Penfield (1958, 1978), was recently revived by Merker (2007). Like GW theory and IIT, Merker’s proposal emphasizes the functional importance of integration – in this case, of external perception, internal motivation, and action selection – but it identifies the core mechanism of integration as an evolutionarily ancient one, centred in the brainstem and subsequently elaborated, in some lineages, to include cortical contributions. Merker appeals to a range of human and animal evidence for subcortical contributions to consciousness, including the claim that hydranencephalic human children possess consciousness despite largely lacking cortical tissue (Aleman and Merker, 2014). To be sure, the brainstem hypothesis is a distinctly minority view in modern human NCC research; upper brainstem activity is more commonly seen as an “enabling condition” for, rather than an immediate correlate of, conscious experience (Koch, 2004). Nonetheless, the current evidence does not definitively exclude the possibility that the upper brainstem is integrally involved in, perhaps even sufficient for, certain forms of consciousness. Within the animal neuroscience literature, (Panksepp, 1982, 1994, 2005; Panksepp et al., 2017) most notably concurs with Merker’s subcortical view of consciousness, especially affective consciousness, although the primary region of interest he identifies is the periaqueductal gray (PAG) of the midbrain. Naturally, such theories which localize the NCC to the brainstem and other non-cortical regions would suggest an especially sweeping distribution of animal consciousness. For example, Barron and Klein (2016) argue that insect brains share structural and functional similarities to vertebrate midbrains and hence may also confer consciousness (for further discussions of invertebrate consciousness, see also Edelman et al., 2005; Feinberg and Mallatt, 2016; Godfrey-Smith, 2017; Mason, 2011; Mather, 2008; Sherwin, 2001).

#### 4.1.5 Translating NCC research to animals

The above sketch of NCC proposals, and the implications and questions they suggest for animal consciousness, is necessarily incomplete. The merits and demerits of each theory continue to be debated, and it is not the goal of this paper to arbitrate between them. Also, we have not discussed popular theories which associate consciousness of perceptual content with circuits of recurrent processing along the ventral stream (Lamme, 2006), or other accounts which suggest alternative functional (e.g., attended intermediate representations; Prinz, 2012) and neural (e.g., the claustrum; Crick and Koch, 2005; Koubeissi et al., 2014) correlates of consciousness. Nonetheless, while far from exhaustive, this brief review suggests some general principles which are broadly relevant to the investigation of animal consciousness in general and affective consciousness in particular.

First, NCC theories often propose an information processing function and an associated neural substrate. Typical functions involve information integration, working memory, flexible response selection, coherent behavioural control, and/or aspects of self-representation. These functions may be characterized in relation to specific mechanisms (e.g., a GW) or more general formal features (e.g., Φ). The neural substrate associated with the relevant function is usually, but not always, assumed to be thalamocortical, perhaps (as in higher-order theories and standard GW theory) with a critical PFC contribution. Second, when a theory proposes a consciousness-associated function/substrate pair, implications for the distribution of consciousness may depend on whether the information processing function or the neural substrate is regarded as critical. Third, it is important to note that neurofunctional theories of human consciousness vary in how far the posited functions are relevant to nonhuman species. In some cases (e.g., the more austere and less permissive variants of higher-order theory (Carruthers, 2000), or perhaps theories which link consciousness to specific theory-of-mind functions that only become critical when social complexity is very high), the functions associated with consciousness may be distinctive to humans, perhaps together with our closest relatives. Other leading theories, however, highlight functions and mechanisms (e.g., relating to information integration and flexible control) with potentially broad relevance to animal cognition, suggesting a far wider distribution of nonhuman consciousness. They also suggest that gradations of consciousness might exist across species, rather than the all-or-nothing presence of full conscious capacities (e.g. see Fahrenfort et al., 2017).

Finally, these general theories of the NCC entail distinctive formulations of the more specific question of conscious *emotion* in animals (on the assumption that emotional and non-emotional contents of consciousness have broadly similar neural correlates; cf. LeDoux and Brown, 2017). Thus, the higher-order theorist would ask whether an animal in a given affective state appropriately represents itself as being in such a state. The GW theorist would ask whether and what affective-state information is broadcast in a (neurally or functionally defined) workspace for the flexible coordination of modular processors. An IIT proponent would ask whether, what, and how affective information is irreducibly integrated into the animal’s neural network. If we knew the right theory of the NCC, we would know the right questions to ask about animal consciousness. In the present state of knowledge, we must be content to make educated but cautious guesses about what the right questions, and hence the possible answers, might be.

### 4.2 Information processing functions associated with consciousness

The search for neural substrates of consciousness has been the most prominent feature of human consciousness research in recent years. But in the animal literature, the potential for identifying information processing functions of consciousness, and the search for parallel functions in humans and non-human animals, have been emphasised. From a comparative perspective, finding the types of information processing in animals that are frequently (if not always; e.g. Persuh et al., 2018) accompanied by self-report of conscious experience in humans has been taken as important suggestive evidence for the presence of conscious experience. To structure this search, it is useful to think of conscious function in humans taking two distinct forms: the *representation* of information in consciousness and the *processing* or cognitive manipulation of information consciously (Shea and Frith, 2016). Conscious *representations* concern the capacity of individuals to not just act on a piece of information, but also to report it as known – i.e., have access to the information processed (see Section 4.2. below for examples of how animals can be trained to “report” information). Such individuals should also show confident expectations regarding the outcomes of actions based on that knowledge. The most studied example of this in humans is conscious vision, although other examples of conscious representations exist, such as metacognition – conscious representations of knowledge. Conscious *processing* in humans involves information processing that can be deliberately controlled: The processing that operates on conscious representations is itself conscious, and hence subject to strategic control. For example, reasoned thoughts and calculations, or something more informal, such as an envisioned ramble through recollections of past events.

#### 4.2.1 Representations and processing in animals

Below, we consider evidence for representations and processing of information in non-human animals that resemble those that are conscious in humans. Phenomena related to conscious vision, metacognition, working memory and episodic memory have all received growing research interest in recent years, with an increasing range of species showing evidence for one or more of these capacities. While none of these studies offer definitive proof that consciousness is involved, they do offer some initial indications of the potential scope of information processing functions of consciousness across the animal kingdom.

##### 4.2.1.1 Vision

In humans, conscious vision, in which an individual reports visual experiences and is confident in their visual judgements, has been compared with blindsight, in which some objective measures of sight remains, but, due to damage to the primary visual cortex, the individual reports no visual experiences and feels that their visual judgements are mere guesses (for reviews see Ajina and Bridge, 2017; Stoerig and Cowey, 1997; Weiskrantz, 1986). The discovery of blindsight was the starting point for much contemporary consciousness research, because it provided, a powerful experimental paradigm in which conscious and non-conscious representations could be compared. It is also a phenomenon that has been extensively investigated in non-human primates, as well as humans, from its earliest days (e.g. Cowey and Stoerig, 1995; Hagan et al., 2017; Humphrey, 1974; Yoshida and Isa, 2015). Dissociating (preserved) visual functions from (absence of) awareness is straightforward in humans where conscious experience tends to coincide with verbal reports used to assess it. However, establishing whether monkeys are also visually aware of the stimuli they respond to is a thornier issue. But so-called “commentary procedures” can allow dissociation between discrimination and awareness to be detected. Results demonstrate that monkeys report ‘no awareness’ only for stimuli in the affected (damaged) visual field, as they classify the very same stimuli they were able to successfully discriminate in a forced-choice task as blank trials when given the option to report whether it is present or absent (Cowey and Stoerig, 1995; Yoshida and Isa, 2015). As a result of such studies, the notion that monkeys such as rhesus macaques possess a capacity for representational consciousness, in the sense of the presence of dissociable features of consciously accessible and non-accessible vision, is scarcely debated by contemporary researchers (Boly et al., 2013). The possibility of a sight/blindsight distinction in other species, however, as well as other similar contrasts in other senses, has been less explored to date (e.g. Carey and Fry, 1993).

##### 4.2.1.2 Metacognition

Another conscious function frequently considered in the search for cognitive markers of consciousness in animals is meta-cognition, the capacity of “knowing that one knows”, or doesn’t know, a piece of information (Smith et al., 1995). For example, in an early experiment also using rhesus macaques, subject animals were required to make a choice between taking a memory test regarding a previously observed image or opting out and not taking the test at all (Hampton, 2001). Subsequent analyses showed that the monkey’s success at remembering was associated with this gamble: they were more often correct when they chose to take the test than when they had to take the test without the opt-out option. Using a range of methodologies, evidence has built for metacognitive capacities across a range of species, including chimpanzees and orangutans (Call and Carpenter, 2001), rhesus macaques (Macacca mulatta, Shields et al., 1997; Hampton, 2001) and rats (Kepecs et al., 2008). In recent years, the mechanisms of metacognition and its putative association with consciousness have been greatly debated (Insabato et al., 2016; Smith et al., 2014, 2016; Terrace and Son, 2009) and continue to be the subjects of active controversy.

##### 4.2.1.3 Episodic and working memory

Several lines of recent research have examined the role of consciousness in memory processes and representations, resulting in proposed cognitive and behavioural markers for conscious memory. These include paradigms for assessing the use of a range of memory representations (including trace vs delay conditioning, delayed matching to sample tasks and episodic memory/episodic future thinking tasks). For example, amongst most humans, the past is not just remembered as if it were a list of facts; recollections are accompanied by reports of rich conscious experiences, like snapshots or video images of past scenes (Tulving, 1985). Damage to or ablation of the hippocampus both eliminates these experiences and severely limits recollection abilities (Rosenbaum et al., 2005). In healthy experimental participants, these episodic memories have been found to carry a number of facets of information content, including the identity, location and timing of an event, leading them to also be described as “what-where-when memories” (Clayton and Dickinson, 1998; Tulving, 1972). And it is these three features of episodic memory that have been sought and found in a number of animal studies, including in birds (Californian scrub jays; Clayton and Dickinson, 1998, 1999) and rats (Crystal and Smith, 2014). However, the mechanisms of what-where-when memories and the question of whether they necessarily require consciousness remain a matter of debate (Crystal, 2018; Mendl and Paul, 2008). For example, it is not yet known whether cases of “aphantasia”, in which people report little or none of the (usually) visual phenomenal content of episodic memories, are associated with functionally reduced capacity to recall what-where-when information (Keogh and Pearson, 2018; Zeman et al., 2015).

Of all consciousness-associated processes observed and studied in humans, working memory and its associated mechanisms has received the greatest scrutiny (see Baddeley, 2003 for review). A number of paradigms have been developed with a view to identifying conscious-like processing in animals by assessing working memory capacities (e.g. Dudchenko et al., 2013). In the hole-board task, for example, animals are given a limited time in which to search an array of holes -in a board for food rewards. The extent to which they avoid returning to previously searched holes, and thereby waste valuable foraging time, is taken as a measure of their capacity to hold very recent events in memory, as occurs in human working memory (Van der Staay et al., 1990). Being able to efficiently forage from an array of potential sites is a skill that can be expected to have been important for the survival of many species, so it is not surprising that a number of mammals have been found to show working memory-like capacities in this task in recent years (mice, Kuc et al., 2006; rats, van der Staay et al., 1990; pigs, Arts et al., 2009). Evidence for other species, including birds, has been found additional tasks, including the matching-to sample task, which has indicated working memory-like abilities in a range of species including pigeons (Blough, 1959; Inman and Shettleworth, 1999; Sargisson and White, 2001; Zentall and Smith, 2016), jungle crows (Goto and Watanabe, 2009), rhesus macaques (Chelonis et al., 2014) and zebrafish (Block et al., 2019; Bloch et al., 2019). However, the extent to which these tasks simulate the complexities of information handling that occurs in human working memory is debateable and if the tasks are testing much simpler processes, the argument that these processes are accompanied by conscious experiences is weakened.

## 5 Conscious affect: what are we searching for?

Having considered correlates of the general capacity for human and animal consciousness, we now focus on the potential correlates of conscious *affect* in particular. To progress in this task, it is first necessary to be clear about the nature and structure of the phenomena that we are searching for – conscious emotional experience. This will allow us to circumscribe the search for conscious emotions to forms that are most likely to occur in non-human animals, and to identify potential complexities in associating measurable indicators with putative conscious experiences of affective states.

### 5.1 The structure of conscious affect: basic emotions, core affect and constructionism

As we discussed in Sections 1 and 3, most emotion theorists take a “componential” view of emotion. Central to this view is the finding that, although they often work in concert, many of the components of emotion are dissociable from one another (e.g. Anderson et al., 2003; Fowles, 2000; Gray, 1994; Kihlstrom et al., 2000; Mammucari et al., 1988; Meadows and Kaplan, 1994). In particular, there is mounting evidence that there is not always a simple, one-to-one correspondence between behavioural or physiological measures on one hand, and self-reported, conscious components on the other (e.g. Barrett et al., 2004; Flack et al., 1999; Gross et al., 2000; Lane et al., 1997; Mauss et al., 2005; Meadows and Kaplan, 1994; Papciak et al., 1985; Reisenzein, 2000; Stone and Nielson, 2001). Some theorists have used this and other evidence to conclude that conscious emotional feelings in humans are not simply the inevitable consequences of the triggering of particular basic or discrete emotional systems, but active ‘constructions’, built as a result of the combination of both top-down (expectation-driven) and bottom-up (stimulus-driven) processing (e.g. see Adolphs, 2017; Wyczesany and Ligeza, 2015). Barrett (2017) argues that, similar to other forms of conscious percept construction such as conscious vision or conscious memory, people do not experience their emotional states as fully pre-formed packages. Instead, they experience bound compounds of (a) core affective experience (e.g. feelings of valence and arousal) and (b) predictive calculations, built on prior experience, of what those feelings might mean and what actions will be needed to respond to them. The Conceptual Act Theory (CAT) of emotion (Barrett, 2014; Barrett, 2017), proposes that emotional experiences are generated via a combination of bottom-up affective and top-down categorization processes (based upon prior experience and mediated by conceptual and linguistic knowledge; Barrett, 2006a, Barrett, 2014; Barrett et al., 2007). According to this constructionist analysis, the correlates of affective consciousness might be expected to come in different guises – as correlates of raw core-affective experiences, and also as correlates of the constructed emotion (e.g. anger, grief), based on a more complex blend of core-affective, cognitive, cultural and linguistic experiences (see Section 5.2.).

From this constructionist perspective, non-linguistic animals would not be expected to consciously experience anything akin to discretely classified emotions in the human sense, whether basic or complex. For example, in response to a question “Does a growling dog feel anger?”, the answer is “…almost certainly no. Dogs do not have the emotion concepts necessary to construct an instance of anger” (Barrett, 2017, p 269; see also Berridge, 2018 for further discussion of this issue). This approach makes a strong distinction between the neural processes that produce emotion-like behaviours in animals (e.g. flee or attack in response to threat) and the equivalent emotions (e.g. fear, anger) as defined, classified, named and experienced by humans (e.g. Barrett, 2017; Barrett et al., 2007; Mobbs et al., 2019). But basic emotion theorists such as Panksepp (1982; 2007; Panksepp and Watt, 2011) have proposed alternative views, claiming that discrete emotions such as sadness, anger and fear are fundamental building blocks of the neural-affective system in animals and humans alike (see also Izard, 2011; LeDoux, 2014b). From this standpoint, the key question is not whether emotions occur at all in non-human species. Rather, it is whether the neural architecture is in place for the expression of such states as conscious experiences. Panksepp insisted that it is (in mammals, at least), proposing that the subcortical structures themselves are able to support affective consciousness (Panksepp, 2005). But this is not a universal view. LeDoux (2014a; LeDoux and Brown, 2017), for example, expects that cortical involvement is likely to be necessary for consciousness of any kind, with various “survival circuits” operating without the necessity of conscious involvement (LeDoux, 2012).

Of course, the notion that there are many distinct forms of emotional experience in humans and potentially animals, is not exclusive to contemporary constructionist theories. For example, appraisal theories have long acknowledged vast arrays of different kinds of emotional experience (Scherer, 2009), as have basic emotion theories (Ekman, 1994; Izard, 1992; Panksepp, 1982). Basic emotion theorists propose that evolutionarily ancient neural programmes or schema can give rise to a relatively small set of complete emotions, incorporating unified suites of feelings, behavioural responses and physiological responses. But these can ultimately result in more or less complex experiences, as a result of cognitive elaboration, or hierarchical classification (e.g. classifying together different basic emotions on the basis of valence; Tellegen et al., 1999). While debate has continued to rage over primacy (Barrett, 2006b; Barrett et al., 2007; Izard, 2007; Panksepp, 2007), there is no question that people can and do report their emotional experiences in a wide variety of ways (Russell, 1991; Yik et al., 2011).

### 5.2 The structure of conscious affect: language, culture, and cognition

Culture, language and cognition all play important roles in defining and characterising facets of human emotional experience (e.g. see Watt-Smith, 2015). While it is important to make a distinction between the processes of *describing* and *experiencing* an emotion (Charland, 2005; Lambie and Marcel, 2002), the issue of how these human-centred processes influence and determine the felt experience is an important problem. And this is especially so for anyone seeking to investigate the possibility of conscious affective states occurring in animals. Which emotions are likely to be uniquely human in nature (or uniquely animal; Nagel, 1974)? And which facets of conscious emotion might we also hypothesise to occur in other species?

Some complex emotions are relevant only to certain individuals or situations and are not discretely recognised across all cultures, let alone outside the human species (Lutz and White, 1986; Russell, 1991; Storm and Storm, 1987). Some others, such as respect, pride or regret may be more commonplace, but may still be associated with syntactic manipulation or linguistic labelling (Johnson-Laird and Oatley, 1989; Shaver et al., 1992). And some are also dependent on conscious capacities such as episodic memory and episodic future thinking (recollection and anticipation; Mendl and Paul, 2008). Many of these types of complex emotions have not been studied in animals. However, a number of recent experimental studies have been used to investigate whether jealousy-like and envy-like responses might occur in some form in non-human species. These have produced somewhat mixed results, but evidence points to responses indicative of some degree of “inequity aversion” occurring in a range of species (e.g. chimpanzees – Hopper et al., 2014; capuchin monkeys – Brosnan and deWaal, 2003; rats – Oberliessen et al., 2016; ravens – Massen et al., 2015; dogs – Range et al., 2009), and “jealousy” in the domestic dog (e.g. see Abdai et al., 2018; Harris and Prouvost, 2014; McGetrick and Range, 2018; although see also Prato-Previde et al., 2018). Whether or not these responses are accompanied by conscious feelings, is not yet established.

The tendency to anthropomorphize animals and make unwarranted inferences regarding human-like emotions is strong and there is a broad consensus amongst researchers to guard against this. A case in point is guilt. Many dog owners express the belief that their pets can experience guilt (Morris et al., 2008). They report seeing guilty or shame-like behaviour, including flattened ears and a retracted tail posture, when their dog is found to have transgressed in some way (e.g. eaten some human food or taken a child’s toy). But consciousness of guilt requires not only a negative feeling of some kind, but also an understanding that this is the result of an action that has broken an established rule. Using an experimental design that gave dogs the opportunity to eat a desirable treat while their owners were out of the room, Horowitz (2009) found that in fact, dogs do not demonstrate any behavioural responses unique to transgressing a rule (e.g. “leave”; “do not touch”). Some do, however, show “guilt-like” postures and behaviours when they expect to be admonished or punished by a human carer, regardless of any knowledge of transgression (see also Hecht et al., 2012).

Different approaches to the nature of emotion and its fundamental building blocks have pointed to different possibilities regarding the nature and structure of animal affects and emotions, conscious or not. The constructionist view suggests that certain non-linguistic animals might experience something akin to human core affective states (i.e. affective valence and arousal; Barrett 2017). But if one takes basic or discrete emotions to be the fundamental building blocks of all conscious emotional experiences, core affective experiences would either not be registered at all in non-linguistic animals, or would be post-hoc constructions/classifications, requiring some degree of categorical or cognitive processing to occur. Evidence from the animal literature regarding these issues is mixed. There is no doubt that many species show particular behaviours or suites of behaviour (akin to fear, anger, panic, etc.) when presented with affectively salient stimuli (Panksepp, 1982). It has also long been known that such behaviours can be triggered by external stimulation of certain sub-cortical brain regions (e.g. Reis and Gunne, 1965; Stellar and Stellar, 1985; Burgdorf et al., 2000). But there is also evidence in favour of core-affect-like processes operating in animals, for example in the form of distinctive and consistent responses being made to different but similarly valenced stimuli (e.g. transreinforcer blocking: Bakal et al., 1974; Balleine and Dickinson, 2006; Ganesan and Pearce, 1988), learned valenced responses to electrical brain stimulation (Burgdorf et al., 2000) and “optimistic” and “pessimistic” behavioural decisions being induced by a wide range of valenced affective manipulations (Harding et al., 2004; Paul et al., 2005). Perhaps in future, debates regarding the basic building blocks of emotion in animals and humans will become more nuanced, incorporating both basic and core affective structures (e.g. see Izard, 2011; LeDoux, 2014b).

### 5.3 Peripheralist and centralist perspectives on conscious emotion: implications for measurement

Averill (1980, 1994), made the important observation that human affective experiences are likely to arise in more than one manner: we can have feelings *of,* feelings *about* and feelings *like*. The *feelings of* category aligns with peripheralist views of emotion, in which emotional feelings arise as a result of someone sensing or observing their own body reacting emotionally. For example, a person might feel afraid if they sense their heart thumping, their hands sweating and their legs running away (James, 1884; Lange and James, 1922). Embodied conscious affective experiences based on sensations of autonomic arousal, emotive facial expressions and emotion-relevant postures are thus examples of feelings *of* (see Section 6.3.). Strong emotions of many different kinds incorporate these sorts of experiences, which are central to contemporary embodiment theories of emotion, and they fit very well with twentieth century notions of emotion as large-scale physiological and bodily events (e.g. Schachter and Singer, 1962; but see also Duffy, 1957 for an alternative view). This approach has been revitalized in contemporary neuroscience by scholars such as Damasio (1999), Park and Tallon-Baudry (2014); Critchley and Garfinkel (2017) and Barrett (2017), to account for the subjective dimension of perceptual experience, including emotions. These authors propose to root subjective experience in the neural representation of visceral information which is transmitted through multiple anatomical path ways to a number of cortical target sites, including posterior insula, ventral anterior cingulate cortex, amygdala and somatosensory cortex. The “neural subjective frame” is not explicitly experienced by itself but is a necessary, albeit not sufficient, component of perception and is postulated to underlie other types of subjective experiences such as self-consciousness and emotional feelings.

It is worth noting, however, that these ‘feelings of’ and their mapping at the central neural level occur to a lesser extent in milder perturbations of affect, such as minor preferences recruited to make everyday decisions (“Tea or coffee?” “Chocolate cake or Victoria sponge?”). Similarly, they are also less likely to be a feature of affective states that are not associated with active behavioural responses (i.e. more cognitively centred emotions such as remorse and regret). In animals, therefore, such autonomic measures of animal affect may be of most accessible in the study of intense emotion-like states.

Cannon’s (1929) alternative to the James-Lange peripheralist stance characterized emotional feelings as occurring directly as a result of central nervous system activity, and only secondarily giving rise to the many other aspects of emotion, including behavioural responses and physiological arousal. Debate between this *centralist* view and James’ and Lange’s *peripheralist* position has lost much of its vigour (e.g. see Ekman et al., 1983; Levenson, 1992). Neither position on its own tells a complete story, and the contribution of each to human conscious emotional experience varies between individuals and circumstances (e.g. see Burriss et al., 2007; Dalgleish, 2004; Wiens et al., 2000; Wiens, 2005). Instead, the centralist position can be seen as aligning most closely with a different facet of felt emotion: Averill’s ‘*feelings about*’ category. Jung (1971/1921); Jung, 1971 talked about emotions as “evaluations of situations or objects”: *feelings about* things such as a delicious meal or a reviled piece of music (see also Berridge and Robinson, 2003; Berridge et al., 2009; Scherer, 2005a,b; Zajonc, 1980; regarding liking and preferences). These are valenced affective judgements, in which the value of a situation or stimulus is consciously registered: positive or negative, pleasant or unpleasant, rewarding or punishing. Perhaps these feelings can be the most prominent of all the facets of affective experience.

A potential proxy measure of these “feelings about” in animals may be the assessment of animal choices and preferences (e.g. Engel et al., 2014; Kirkden and Pajor, 2006). However, we should be aware that choice can be effected in a number of different ways. For example, preference in the context of multiple choices can be quite different to liking or disliking an individual stimulus. Choices in value-based decision paradigms can be driven by Pavlovian, habitual, or goal-directed processes that may vary in how closely they reflect consciously experienced pleasure or displeasure. Formal investigations of whether and how positive and negative feelings in humans are associated with these different kinds of evaluation would help inform their use as indicators of conscious affect in animals.

*Feelings like* concern anticipated instrumental responses, such as “I felt like running away” or “I feel like crying”. These types of conscious affective experiences correspond to a view of emotions as preparatory action sets or tendencies (Frijda, 1986; Öhman et al., 2000; Panksepp, 1994). Instead of being to do with a feeling of the body acting, they are feelings associated with preparations or intentions to act, and are likely to result from interoceptive sensations of the commencement of the action (i.e. a peripheral process). They may also involve registration of a form of action motivation, action readiness, or action inhibition, as a centralist process (c.f. action tendencies; Lowe and Ziemke, 2011). Potential measures in animals include anticipatory behaviour occurring in the presence of predictive cues (e.g. Spruijt, 2001). As with *feelings of*, *feelings like* seem to be most associated with strong emotions that incorporate active (or expressive) behavioural responses. Anticipatory actions may thus be useful indicators of these states in animals, but not of other affective states that are so mild or diffuse that they are unlikely to incorporate *feelings like* at all.

In conclusion, the emerging message is that conscious affect is not a unitary phenomenon and so we should be clear about the type of conscious emotion-like states that we are seeking to assess in other species – a simple discrete emotion-like state, a valenced affective state, a cognitively complex emotion-like state. Furthermore, specific indicators may be more likely to reveal one type of conscious affective experience than another – autonomic activation and anticipatory behaviours may be particularly suited to the assessment of more intense *feelings of* and *feelings like* affective states, whilst choice and preference may tell us more about *feelings about* states.

## 6 Neural correlates of affective consciousness

With the details of Section 5 in mind, we now adapt the NCC approach outlined in Section 4 to consider neural correlates of *affective* consciousness (NCACs). Affective states in humans have been associated with the involvement of numerous cortical and subcortical brain regions, including the prefrontal cortex, insula, cingulate cortex, amygdala, thalamus, nucleus accumbens, ventral pallidum, periaqueductal grey, etc. (e.g. Davidson et al., 1999; Phan et al., 2002). However, much remains unresolved regarding the contributions of specific areas to conscious vs. non-conscious components of emotion. In this section, we first revisit the NCC theories discussed in Section 4. From the vantage point of each theory, we ask what shape the NCACs would be expected to take, and how, if the theory is accepted, conscious emotional contents could in principle be inferred from neural data.

We then turn from principle to practice, considering theoretically less pristine but empirically more tractable methods which may help guide the search for NCACs in the near term. We focus on the different types of study that may help us address this issue: for example, studies of individuals with brain injuries or other pathologies which render them incapable of feeling emotions, even though they may nevertheless be able to “do” emotions in other respects (Section 6.2), and studies of non-clinical, naturally occurring, between-subject variation in people’s ability to detect the conscious features of their emotions (Section 6.3). Finally, in Section 6.4, we consider the implications of these findings for animals.

### 6.1 General theoretical considerations

In Section 4.1, we outlined several influential theories of the NCC, each of which associate consciousness with an information processing function implemented in a particular neural substrate. Even if these theories don’t explain *why* the function/substrate in question is accompanied by experience (a question some take to be beyond the reach of human understanding; DuBois-Reymond, 1874; McGinn, 1989), they nonetheless furnish candidate empirical criteria for both the identification of consciousness and the decoding of its contents. That is, if we knew that one of the current NCC theories were correct, and if our capacity to measure neural activity were unconstrained, the theory would give us a recipe for reading off the information content of consciousness from observations of the brain. If the theory applies to all conscious contents, it would, in particular, allow us to measure the affective contents of consciousness – i.e., whether it includes information related to the valence and to the motivational meaning of internal states and/or external objects. Translating to animals, such an ideal read-out might not illuminate what it is like (in the sense of Nagel, 1974) to have the organism’s emotional experience, but it would specify what, if any, affective information was consciously experienced, as well as the relationship of that affective code to other consciously available information (e.g. interoceptive sensation of heartbeat, etc.).

Of course, there is no accepted consensus as to which, if any, of today’s NCC theories is on the right track, and existing measures of neural activity are far from ideal. Thus, in practice, we must make do with tentative interpretations of data that are invariably imperfect, though improving in quality at an accelerating pace (see Section 6.2). Still, it is instructive to consider what, under ideal conditions, the empirical signatures of conscious emotion *would* look like according to currently influential theories of the NCC and how these might be sought in animals.

For theories of the NCC focusing on information integration and/or flexible behavioural coordination (i.e. ITT and GW theory; Baars, 1988; Dehaene and Naccache, 2001; Tononi, 2008; Tononi et al., 2016) the question assumes the following form: What, if any, affective information is integrated to a sufficient degree and/or in the theoretically relevant behaviour-guiding way? Note that this functionally integrated information could be continuous (*à la* variations in core affect) and/or categorical (*à la* basic emotions or more complex cultural-linguistic constructions). But importantly, on any theory of this general kind, one would expect conscious contents to have some kind of “affective tone,” albeit one that could differ widely across species. For if information is integrated for the flexible control of behaviour, it should presumably encompass not only perceptual items, but also (in one way or another) their motivational significance for the organism – i.e., the valence of perceptual affordances in relation to (possibly fine-grained aspects of) the organism’s present internal state and behavioural goals.

Particular theories imply more specific procedures for decoding the contents of consciousness. In GW theory, the ideal measurement strategy would be remarkably straightforward: Simply eavesdrop on the brain’s (limited-capacity) for global broadcast. For animal brains with attention and executive control networks resembling ours, this would presumably involve recording and decoding the outputs of frontoparietal neurons with wide cortical projections (Dehaene and Naccache, 2001; van Vugt et al., 2018). The question of conscious emotion would then reduce to the question of whether and how signals in the putative frontoparietal broadcast encode stimulus valence in relation to affective behavioural control (emotional and motivational consciousness).

The ideal measurement of conscious emotion is less conceptually simple in IIT, because it associates consciousness with a general network property (Φ) rather than a specific computational mechanism, and because of the practical challenges in computing Φ for all but the simplest systems. Yet if, computational barriers notwithstanding, a general “Φ-meter” for information networks could somehow be devised, the contributions of different emotion-related areas to the brain’s “main complex” (Tononi and Sporns, 2003), and hence to the animal’s conscious experience, could in principle be measured. Perhaps more realistically, but still ambitiously, if methods of assessing effective connectivity (e.g., Massimini et al., 2005; Rosanova et al., 2012) are sufficiently enhanced, it may eventually be possible to more finely characterize how precise local perturbations of specific emotion-related areas (e.g., amygdala vs. insula) as well as other areas (e.g., primary visual vs. inferior temporal cortex) do or don’t globally propagate across the cortex, an operational measure of information integration.

From the perspective of higher-order theories (HOT and HOE: Lau and Passingham, 2006; Lau and Rosenthal, 2011), the authentic neurofunctional signature of conscious emotion should be some form of emotional metacognition (“metaemotion”), presumably implemented in circuits prominently involving the dorso-lateral PFC. But the details will depend on which of the numerous variants of higher-order theory one adopts – some of which require the self-application of a sophisticated theory of mind (e.g., Carruthers, 2000) while others focus on simpler forms of metacognition (e.g., calibration of behaviour to evidential confidence; Lau and Rosenthal, 2011). Animals that are currently known to show such capabilities are primarily mammalian.

### 6.2 Neural correlates of ‘doing’ and feeling emotion

In the search for neural correlates of perceptual consciousness, researchers have generally employed the contrastive method to compare neural activity in apparently matched cases of conscious vs. non-conscious perception. It is natural to employ a similar strategy in the search for NCACs, contrasting neural concomitants of conscious vs. unconscious affect.

In seeking to make this contrast, it is important to draw a distinction between the unconscious perception of emotional stimuli and the unconscious occurrence of emotional or affective states themselves. The first focus is comparatively straightforward, and a wealth of human research has identified neural and behavioural responses to affective properties of reportedly unseen visual stimuli. For example, subliminal fearful expressions and emotion words spur heightened amygdala activity (e.g., Naccache et al., 2005; Whalen et al., 1998; Tamietto and deGelder, 2010), and subliminal reward-predictive symbolic cues can apparently drive instrumental conditioning, with associated activity in the amygdala and ventral striatum (Pessiglione et al., 2008). Similarly, patients with damage to primary visual cortex sometimes exhibit blindsight not only for simple perceptual properties like location and line orientation, but also for emotional facial expressions and body postures (Pegna et al., 2005; Tamietto et al., 2009; Celeghin et al., 2015; De Gelder et al., 1999; Burra et al., 2019; Bertini et al., 2013). Unseen emotional stimuli in this kind of “affective blindsight” induce spontaneous facial mimicry or physiological arousal and have also been linked to activity in a subcortical pathway relaying non-consciously processed visual information to the amygdala (Morris et al., 2001; Pegna et al., 2005; Tamietto et al., 2009; Tamietto et al., 2012). Unconscious perceptual analysis, associated with activity in relevant local circuits, thus appears to extend to the coding of affectively significant stimulus properties.

Turning from stimuli to states, a natural strategy for NCAC research is to search for unconscious emotional-state syndromes – i.e., cases in which multiple (behavioural, physiological, etc.) components of an emotion are demonstrated without any actual *feeling* of a conscious emotional state. When subliminal emotional stimuli support learning (e.g., Pessiglione et al., 2008) or generate autonomic reactions (e.g., Gläscher and Adolphs, 2003), it is tempting to infer that components of emotional response must be occurring in the absence of consciously felt emotion. Note, however, that this inference assumes that the affective properties of unseen stimuli leave no trace on global conscious affect. While not implausible, this assumption is rarely directly tested in studies of unconscious perception of affective stimuli (although see Winkielman et al., 2005 for an example of a behavioural paradigm which specifically examines this assumption).

Frontal brain injuries have been documented to be associated with a number of significant emotion-related deficits, including difficulties in emotional control, and in the anticipation of future positive and negative emotional states (e.g. Bechara et al., 2000; Engberg and Teasdale, 2004). However, as yet, no comprehensively documented clinical cases have linked specific lesion sites to unconscious emotion syndromes, although the possibility of such unconscious emotion has been discussed theoretically (e.g. Kihlstrom et al., 2000; Lane et al., 1997) and some candidate cases have been reported (e.g. Nielsen et al., 2000). Plum et al. (1998) describe a patient with a vegetative state diagnoses, clinically considered to have no (perceptual, emotional, or other) consciousness, but who nonetheless exhibited extreme stereotyped rage reactions upon stimulation, with multiple behavioural and autonomic components (e.g., screaming, clenched teeth, and elevated blood pressure). The researchers attributed this putative emotional automatism to the isolated operation of a network of relatively active right hemisphere cortical and subcortical areas, against a backdrop of more globally depressed cerebral metabolism.

Biraben et al. (2001) found that epileptic seizures which involve the amygdala, anterior cingulate cortex and prefrontal cortex are often accompanied by subjective feelings of fear, while comparable seizures which involve the amygdala alone are not associated with fearful feelings. These findings concur with others that have shown that amygdala damage is not associated with deficits in the subjective experience of emotion (even though some behavioural and physiological responses are impaired) (e.g. Anderson and Phelps, 2002; although see also Feinstein et al., 2011 for contrary results), while anterior cingulate cortex (ACC) damage has been found to significantly impair conscious affective experiences (Phan et al., 2002). So, although both structures have clear importance in the processing of emotion, only the ACC may be necessary for certain facets of conscious emotional experience.

The orbitofrontal cortex (OFC) and ventromedial pre-frontal cortex (vmPFC) are well known to be involved in the valuation of both primary and secondary reinforcers in humans (e.g. the pleasantness of food and more abstract reinforcers such as money or music) and patients with OFC and vmPFC lesions demonstrate significant abnormalities of affective decision making (see Chib et al., 2009; Kringelbach and Rolls, 2004). This frontal region has been suggested by some to be important in the generation of conscious emotion components such as affective valence in humans (e.g. see Kringelbach and Rolls, 2004; Kringelbach, 2005). But evidence from OFC damaged patients has not offered a clear case for non-conscious affect. People diagnosed with developmental psychopathy, a condition also associated with frontal-cortical abnormalities, appear to show an almost opposite syndrome: verbally reporting emotions such as fear, yet exhibiting little or no behavioural or physiological fearfulness (Herpertz et al., 2001; Patrick et al., 1993, 1994).

Another area relevant for attention and consciousness is the parietal cortex (PC). Damage to the right PC often causes hemispatial neglect, wherein patients do not represent and react to stimuli in the contralateral side of the lesion. However, emotional (typically fearful) expressions presented to the neglected side of the space tend to summon attention and to be consciously detected more often than neutral stimuli (Vuilleumier et al., 2002; Vuilleumier & Schwartz, 2001; Williams & Mattingley, 2004; Tamietto et al., 2007, 2015). Consciously perceived emotions are uniquely associated with activity in areas that map somatic (i.e., visceral and somatosensory) changes, such as the insula and somatosensory cortex (see Section 6.3. below).

Finally, the relationship between “doing” emotion or affect (i.e., enacting emotional expressions and behaviour) and subjective feelings has been addressed directly in a series of studies applying intracortical electrical micro-stimulation to patients with pharmacologically resistant epilepsy (Caruana et al., 2015; Caruana et al., 2018). Electrical stimulation of the ACC in the pregenual sector (pACC) elicited bursts of laugher that in half of the subjects was also accompanied by conscious positive affect (Caruana et al., 2015). Likewise, electric stimulation of the amygdala evokes visceromotor responses typical of response to threat that are also accompanied by subjective feelings of fear (Meletti et al., 2006), akin of that observed for anterior insula and disgust (Krolak-Salmon et al., 2003). These findings demonstrate at the micro-scale level of a few limited neurons, that expression and experience of emotions actually map into a common neural code that helps situate agency as a core feature of emotional experience. However, a more recent study has challenged this conclusion with observations that precise amygdala stimulation can increase electrodermal activity and heart rate, without eliciting any changes in conscious experience (Inman et al., in press).

### 6.3 Neural correlates of individual variation in emotional awareness

Individual variation in conscious emotional awareness has been studied across a number of different strands of research. One to receive particular attention in the clinical and psychosomatic literature in particular is the study of *alexithymia*. Alexithymia is a variable trait within the normal population, comprising reduced emotional experience and difficulty in recognizing and labelling emotions (Bagby et al., 1994; Lane et al., 1997; Smith et al., 2019; Lane et al., 2015; Stone and Nielson, 2001). It has high levels of co-morbidity with neurodevelopmental disorders such as autism and is associated with depression and a range of somatising disorders (Li et al., 2015; Honkalampi et al., 2019). Alexithymic symptoms have also been found among cerebral commissurotomy patients, prompting the suggestion that difficulty recognizing and describing emotional feeling states may result from inadequate connections between the right, “emotional hemisphere” and the left, “linguistic hemisphere” of most human brains (Hoppe and Bogen, 1977). Some studies have supported such interhemispheric theories of alexithymia, showing that alexithymic individuals tend to have some degree of transfer deficit between the two sides of the brain (e.g. Romei et al., 2008; Zeitlin et al., 1989). But not all studies have confirmed this (e.g. Grabe et al., 2004), and alternative hypotheses have been proposed (e.g. Lane, 2008; Tabibnia and Zaidel, 2005). Most prominently, Lane (2008) suggested that alexithymia may be an abnormality of the vertical rather than horizontal axis of the human brain, resulting from inadequate development of neural connections between the evolutionarily “older” emotional centres (brainstem, diencephalon, limbic system) and more recently evolved regions (paralimbic, prefrontal cortex). This, he argued, might lead to reduced conscious awareness of ongoing (sub-cortical) emotional processes and less top-down regulation of them. A number of studies of the brain activity of normal individuals varying in emotional awareness have provided some support for these ideas. Using positron emission tomography (PET), Lane and colleagues (Lane et al., 1998) found that higher levels of emotional awareness in women (i.e. greater ability to detect and classify emotional feelings; Lane et al., 1990) were associated with greater regional cerebral blood flow in the dorsal anterior cingulate cortex (dACC) when participants recalled emotional events or viewed emotive film clips. McRae et al. (2008) found a similar correlation between levels of emotional awareness and blood flow in the dACC, but on this occasion, it was clear that the association was dependent on the arousal qualities of the emotional stimuli being viewed, but not their valence (i.e. the correlation only occurred when participants, males and females, viewed highly arousing emotive pictures; no differences in dACC blood flow were found for positive vs negative pictures). Mantani et al. (2005) found reduced posterior cingulate cortex activation during emotional imagery tasks in alexithymic individuals. And more recently, meta-analytic studies have found evidence for associations between alexithymia and functional alterations during emotion processing in the amygdala, insula and medial prefrontal cortex (van der Velde et al., 2013) and structural alterations (reduced volumes) in the left insula, left amygdala, orbitofrontal cortex and striatum. Taken together, these studies suggest heterogeneous and predominantly cortical deficits in alexithymia (see also Xu et al., 2018). However, the extent to which these findings are associated with conscious emotional feelings *per se*, or with the capacity to name and put words to those feelings, is not yet clear (Aleman, 2005).

Research into individual variation in conscious *interoceptive* experience has tended to be largely independent of studies of alexithymia. If much or all of our emotional feelings arise from sensed physiological changes inside the body, it follows that an ability to monitor bodily responses is an important pre-requisite of a capacity to consciously experience emotion. Certainly, people’s verbal descriptions of their emotions often include descriptions of conscious interoceptive experiences (Nummenmaa et al., 2014). The term interoception refers to the multitude of processes by which the internal states of the body (heartrate, blood pressure, blood glucose levels, gut activity, inflammation, etc.) are monitored, and is closely associated with peripheralist views of emotion. Currently a rapidly advancing field of research (e.g. see Critchley and Garfinkel, 2017; Khalsa et al., 2018; Pace-Schott et al., 2019), numerous interoceptive systems have been identified in humans, involving a range of afferent pathways (for reviews see: Craig, 2004; Critchley and Harrison, 2013; Strigo and Craig, 2016). Of course, not all of these diverse processes of interceptive sensing necessarily result in consciously felt sensations or emotions. Many contemporary accounts of the role of interoception in emotion incorporate Bayesian conceptions of predictive coding (Barrett and Simmons, 2015; Seth and Friston, 2016). These point to a major function of interoception being one in which predictions, data acquisition (through interoceptive sensing) and prediction error signal generation produce bodily responses which maintain homeostasis. And this may often occur without the need for any kind of conscious emotional involvement (e.g. via reflexive and humoral action). Conscious sensing of the internal melieu of the body, such as heartbeat detection, and the conscious emotional sequelae of this, can be hypothesised to function to achieve homeostasis in two ways. First, it can act by motivating or guiding behaviourally appropriate responses (e.g. moving from a sunny to a shady location to prevent overheating), and second, by enabling individuals to prioritize urgent behavioural responses (e.g. fleeing from threat) over ongoing physiological, non-conscious, allostasis (McEwen and Wingfield, 2003; Pace-Schott et al., 2019). Of note here is the finding that, at the neural level, vertical processes, similar to those identified within alexithymia research, are involved in conscious interoceptive sensing and emotion (although see Nicholson et al., 2018). For example, arterial baroreceptor-mediated heartbeat detection leads to activations within the insula cortex and has also been found to have rapid and measurable effects on both conscious affective experience and cognitive-affective processing (Critchley and Garfinkel, 2017; Garfinkel and Critchley, 2016; Garfinkel et al., 2014; Strigo and Craig, 2016). While further research into the neural correlates of individual differences in accurate and consciously registered interoception is still required (e.g. see Garfinkel et al., 2015), the role of cortical regions (posterior, mid and anterior insular cortex, cingulate and pre-frontal cortices) and their interaction with sub-cortical regions (amygdala and striatum) are currently the primary focus (see Garfinkel and Critchley, 2016 for recent review).

### 6.4 Implications for animals

The validity of translating knowledge of neural and functional correlates of affective consciousness in humans to other animal species will inevitably be constrained by the degree of structural and functional similarity between their brains and ours. Research in this field is still in its infancy, but examples do exist. Demonstrating that lesions to the medial prefrontal cortex (mPFC) in rats resulted in impaired decision-making in a rat variant of the Iowa Gambling Task, van den Bos et al. (2014) also showed that these impairments were similar to those observed in humans with lesions of the vmPFC, indicating some functional and anatomical equivalence. The role of conscious affect in such tasks has been discussed at length in human studies, and it is possible to speculate that damage to the mPFC interferes with decision-making in both species via disruptions to conscious experience of affect or interoception (e.g. “somatic markers”; Damasio, 1999). However, Damasio and colleagues have been equivocal as to whether conscious emotion is necessary to act as a ‘somatic marker’ in this kind of decision-making; in future, clearer human models of conscious vs non-conscious affect are likely to prove a more appropriate starting point (see Section 6.2).

A different approach has been to use manipulations of brain areas that, in humans, are associated with conscious affect, to identify behaviours that may be potential markers of equivalent, conscious affect in animals. For example, Langford et al. (2010) studied facial ‘grimace’ expressions as indicators of pain in mice. They found that damage to the rostral anterior insula, an area of the brain implicated in human conscious experience of the affective unpleasantness of pain, resulted in attenuation of grimace expressions but not of other behaviours such as abdominal constriction. They speculated that this might indicate the utility of grimaces, but not abdominal constrictions, as markers of conscious pain as opposed to just sensory nociception. Of course, such reasoning requires one to accept that, as in humans, the rostral anterior insula also plays a role in conscious affective pain in mice.

To move forward in this area, it will be important to consider the practical and theoretical backgrounds from which to approach comparative investigations of consciousness and conscious affect in humans and a range of non-human animals. Amongst human observers, there is tremendous individual variation in beliefs about animal consciousness and sentience (capacity to consciously experience feelings and emotions; Phillips and McCulloch, 2005; Spence et al., 2017). But there is also a strong agreement and emphasis on a phylogenetic distribution of conscious capabilities across the animal kingdom, with smaller and smaller-brained species (invertebrates, fish, amphibians, etc.) being regarded as less intelligent and less sentient than larger and larger-brained ones (e.g. mammal, primates; Clarke and Paul, 2019; Kupsala et al., 2016; Nakajima et al., 2002; Walker et al., 2014). And this view is recapitulated in much legislation, with primates, mammals and vertebrates being given most legal protection (Home Office, 1986). However, such phylogenetic approaches have significant limitations and are inevitably vulnerable to anthropomorphic biases (Dacey, 2017).

Within the realm of comparative cognition, the questions of whether and to what extent animals differ in cognitive capacities such as general intelligence, learning ability or specific skills, such as spatial memory, have been studied and discussed extensively (Zentall, 2013). Both inter- and intra-specific studies have provided support for a connection between brain size (including brain size corrected for body size) and cognitive capacity (Buechel et al., 2018; Horschler et al., 2019; Kilmer and Rodriguez, 2019; Kotrschal et al., 2013; Overington et al., 2009). There is also evidence for connections between the size of particular brain regions (e.g. PFC and hippocampus) and specific cognitive capacities (social intelligence and spatial memory – Dunbar and Shultz, 2007; Hampton and Shettleworth, 1996). But qualitative as well as quantitative differences between the brains of animals from different phylogenetic groups make such comparisons between species highly problematic. For example, mammalian primate and non-primate cortices differ in a number of important respects, including volume/neuron density ratios (Herculano-Houzel et al., 2007, 2012, 2017). And bird and mammal brains differ radically, with the avian nidopallium caudolaterale (NCL) displaying few anatomical similarities to the mammalian PFC (Güntürkün and Bugnyar, 2016). Nevertheless, it has proven surprisingly difficult to conclusively demonstrate extensive and systematic variation in cognitive capacities between such groups of animals (Pearce, 2008). While some differences certainly do exist, there is a growing body of evidence showing that cognitive capacities can transcend phylogenetic boundaries, even including the vertebrate – invertebrate distinction (e.g. Japyassu and Laland, 2017; Schnell and Clayton, 2019; Simons and Tibbetts, 2019 for description of cognitive skills in spiders and other invertebrates). In sum, it is now widely accepted that variation in the cognitive capabilities of animals can be attributed as much to adaptations to ecological need (e.g. dependence on stored food –e.g. see Grodzinsky and Clayton, 2010; Shettleworth, 2012) as to differences in phylogeny, brain structure or brain size. Consequently, the argument that smaller and smaller-brained species are necessarily less cognitively sophisticated, and hence less likely to require conscious processing, is undermined. Behavioural ecological needs may better predict variation in these capacities across species, and any associated capacities for affective consciousness.

The theories of consciousness reviewed here (Section 4), and the neural and functional correlates of consciousness, affective consciousness and emotion (described in Sections 5 and 6) have largely, though not exclusively, focused on cortical involvement, and interactions between cortical and subcortical (amygdala, thalamus) regions in humans. As a consequence, animals that share homologues of human structures such as the PFC, insula, ACC and parietal cortex are important candidates for future comparative research into structural and functional correlates of potential correlates in animals. But contemporary approaches in the study of comparative cognition outlined above point to the value of investigating potential correlates of affect and consciousness across a wider range of species. Evolutionary theories posit both generalised cognitive capacities across a wide range of animals (e.g. habituation, simple associative learning), coupled with specialised cognitive skills advantageous within particular ecological or behavioural niches (e.g. spatial memory; episodic future thinking; Pearce, 2008; Shettleworth, 2012). If parallel processes apply to the evolution of consciousness, we are led to consider whether consciousness (and its variants, including conscious affect) is the product of specialization or generalization. That is, is it likely to be a process that facilitates a widespread function (e.g. decision-making) across many phyla and species, through phylogenetically preserved neural structures? Or is it more specialised process, useful in only a limited range of behavioural niches? If the latter, parallel evolution of conscious processing may be found in some distantly related species that share decision-making challenges.

## 7 Cognitive and decision-making correlates of affective consciousness

The functional or information processing correlates of consciousness approach outlined in Section 4 can also be focused specifically on affective states. An important proposed function of conscious affect is in guiding decisions and, accordingly, the present section focuses on information processing functions of conscious affect in the form of decision-making and choice.

### 7.1 Decision-making functions of affect in humans

A prominent hypothesis for a function of conscious emotional experience in humans is that felt emotional experiences act as pieces of information, to be made use of when judgements or decisions are made (e.g. Damasio, 1994; Forgas and Bower, 1988; Forgas, 1995; Frijda, 1986; Gasper and Clore, 1998; Lerner and Keltner, 2000, 2001; Loewenstein et al., 2001; Pham, 1998; Schwarz and Clore, 1983, 1988, 2003, 1996; Wyer and Carlston, 1979; Zillman, 1978). These ideas bear close similarities to theories of consciousness such as the global workspace proposals of Baars and colleagues (e.g. see Baars, 2002; Newman and Baars, 1993), summarized above. Specifically, both global workspace and affect-as-information theories suggest that an information processing advantage is gained by using conscious representations to bring together the disparate neural systems involved in different facets of cognition and emotion (see also Morsella, 2005).

The core psychological idea of affect-as-information theories is that referring to one’s immediate, felt, emotional state can act as a quick, heuristic processing method for making judgements, particularly in the case of complex, urgent, ambiguous or uncertain decisions. Affective states that have not been assigned to a specific cause or origin (i.e. free-floating feelings or moods that the experiencer has not consciously attributed to a particular cause or attached to an object) are the most likely to be used to influence judgements in affectively congruent ways. In their seminal study, Schwarz and Clore (1983) telephoned hundreds of participants about their general satisfaction with life; participants were more likely to report that this was high when the weather was sunny. However, if the pleasant weather was brought to their attention, satisfaction ratings were no longer elevated (but see also a recent debate about the size, boundary conditions and robustness of this effect – Schwarz, 2017; Yap et al., 2017).

Similar ideas have been applied to the fields of neuroeconomics and decision-making under risk. For example, in his risk-as-feelings hypothesis, Loewenstein argued that people make use of emotional feelings to calculate the value and probability of the potential outcomes of risky gambles and other economic decisions (Loewenstein et al., 2001, 2008; cf. Slovic and Peters, 2006). Sometimes these feelings may represent ongoing moods, while on other occasions, they may reflect moment by moment changes in core affect, with, for example, wins and losses in a gambling game influencing subsequent decisions (e.g. Croson and Sundali, 2005). And in the field of clinical neuroscience, Damasio (1994) proposed that people attend to their peripheral, somatic (including autonomic and enteric) responses to emotive or risky situations in order to make decisions about the best course of action. Evidence for this comes from individuals with orbitofrontal damage, who often make disadvantageous decisions, perhaps because of an inability to generate somatic markers pertaining to decision outcomes that may be punishing, such as the loss of money (e.g. Bechara et al., 1997, 2000).

Here we should note that whether or not such processes are exclusively dependent on consciously experienced emotions is not yet certain. Proponents of the somatic marker hypothesis claim that anticipatory somatic responses could be used both consciously and unconsciously (i.e. explicitly and implicitly) to guide decisions (e.g. Bechara et al., 2005). And it has not yet been conclusively demonstrated that all such decisions are necessarily reliant on consciously experienced affective information. Indeed, Winkielman et al. (2005) showed that some decisions appear to be influenced by affective reactions that people do not consciously report. They induced ‘unconscious’ emotional states in human participants by showing them subliminal images of smiling and frowning faces (see Mogg et al., 1993; Niedenthal, 1990; Zajonc, 2000). Thirsty participants who were exposed to happy as opposed to angry faces, poured and drank more of a novel sweet drink, and reported that they were willing to pay significantly more money for it. Winkielman and colleagues concluded that this was an example of unconscious emotion – an affective response powerful enough to influence preference/liking related behaviour, but not registered consciously by participants as an actual felt state. Winkielman and Gogolushko (2018) found similar results with both submiminal and supraliminal facial expression primes, though not with emotive words presented subliminally and supraliminally (which produced incongruent effects). Although there is significant support for the notion that consciously experienced affective states guide human decision-making, we should thus be aware that such effects may also proceed via routes that do not require conscious experience of emotion.

### 7.2 Implications for animals

The idea that affective processes play a key role in decision-making opens up possibilities for studying choice behaviour as a potential marker of animal affect and even conscious emotion – a NCAC. Here we briefly consider decision-making scenarios where a role of affect has been proposed in animals: simple trade-off choices between different types of resource; affective influences in reinforcement learning; and affective influences on decision-making under ambiguity.

A number of researchers have considered the possibility that affect may be involved in adaptive decision-making in animals, especially where disparate motivations are pitted against one another. The focus of these proposals is on prospective and anticipated affect and its role in risky and value-based judgements. Cabanac (1992) argued that animals make choices between different sorts of resource (e.g. food, water, sweet taste, temperature) in an additive way that can be accounted for by the existence of some form of ‘common currency’ with which to weigh up the relative rewards expected from each resource. He proposed that the consciously experienced affective state of pleasure fulfils this role, and functions to arbitrate in behavioural decisions that require the comparison of different sorts of reward or punisher. Niel and Weary (2007) investigated similar trade-offs in rats to establish the relative aversiveness of different types of gases used for euthanasia (e.g. CO_2_), with subjects being required to choose between consuming highly palatable food rewards and spending time in a gas-perfused chamber (see also Cooper et al., 1998). Studying a very different species, Elwood and Appel (2009) investigated trade-off decisions between reward and punishment in hermit crabs. These animals show strong preferences for moving to larger discarded mollusc shells in which they live. But when a large shell also delivers electric shocks over a certain voltage, they prefer to move to a smaller one. There are of course many other examples of trade-off decisions that animals face daily. It is possible that optimal decisions in such situations require conscious experience of emotion but equally that non-conscious affective processes are at play. A step towards resolving these alternatives is to develop experiments in humans that confirm or refute the hypothesised link between conscious affective experience and the capacity to engage in these sorts of trade-off decisions.

Another area of conjecture regarding the functions of conscious affect in animals concerns the possibility that it has one or more roles in reinforcement learning. From the earliest days of experimental animal psychology, it was realised that affect (in the human sense of pleasure and displeasure) bears close parallels with behavioural reinforcement and punishment (Thorndike, 2011). However, it is of course possible that any affective processes involved are non-conscious (e.g. Damasio, 1999; LeDoux, 1996). For example, Berridge and colleagues (e.g. Berridge, 2000; Berridge et al., 2009; Berridge and Winkielman, 2003) have proposed that the sub-cortical brain network that gives rise to ‘core liking’ reactions in rodents to stimuli such as sweet tastes (i.e. behavioural responses such as lip-licking) needs to interact with cortical brain systems if the conscious registration of such states is to occur. Species that do not have this capacity may perform affective or hedonic behaviours, and learn as a result of reinforcement, but not actually experience affective feelings akin to human liking or disliking. Birds, reptiles, decerebrate mammals, and even invertebrates such as aplysia have been found to show reinforcement learning (Bromily, 1948; Burghardt, 2013; Emery and Clayton, 2004; Norman et al., 1977; Walters et al., 1981). So a cerebral cortex is certainly not necessary for reinforcers to affect behaviour. And compelling evidence for non-conscious reinforcement comes from numerous studies of learning under anaesthesia. Such learning, including food aversion learning and tone-shock associations, has been demonstrated in adult rats (Roll and Smith, 1972; Rabin and Rabin, 1984; Bermudez-Rattoni et al., 1988; Edeline and Neuenschwander-El Massioui, 1988), foetal rats (Mickley et al., 1995), mice (Pang et al., 1996) and sheep (Provenza et al., 1994).

Despite these findings, the possibility remains that some forms of reinforcement- learning, such as goal-directed learning, do indeed require consciously experienced affect to occur. Rats can show both a simple form of learning of the association between a cue and the value of a reinforcer (habit or ‘model-free’ learning), and a more complex form of association between a cue and both the value and identity of the reinforcer (i.e. water or food; goal directed or ‘model-based’ learning) (Dayan and Berridge, 2014). It has been suggested that the consciousness of affect may be needed to bring together these two types of information (affective - the value of the reinforcer; cognitive - the identity of the reinforcer), in order to produce successful goal-directed learning (Dickinson and Balleine, 2009). Although computationally more demanding, these types of association confer information processing advantages, allowing animals the flexibility to change and redirect instrumental behaviours according to current needs (e.g. current hunger vs current thirst). But many questions still remain about goal directed learning, including which species can make use of it, and whether, in humans, any conscious affect can be shown to be required for such associations to be formed. More generally, clear evidence that reinforcement learning in humans requires rewards and punishers to be experienced as conscious feeling states (i.e. of positively or negatively valenced affect) would help shed further light on the potential utility of inferring conscious animal affect from reinforcement learning.

Paralleling the affect-as-information hypotheses described above, it has been speculated that some animals may also use affective states to aid adaptive judgement and decision-making when there is uncertainty about decision outcomes. Using tests of decision-making under ambiguity or ‘judgement bias’ (Paul et al., 2005; Mendl et al., 2009a,b, 2010), many studies have demonstrated information processing biases in a wide range of species in response to affect manipulations (e.g. rats - Harding et al., 2004; dogs – Mendl et al., 2010; starlings - Bateson and Matheson, 2007; Ravens - Adriaense et al., 2019; Honeybees – Bateson et al., 2011). In line with findings from humans, subjects assumed to be in an induced positive affective state tend to respond to ambiguous stimuli as if they predict reward, while those in a more negative affective state behave as if they predict a punisher (although there are also some null and opposite findings – e.g. Muller et al., 2012; Ross et al., 2019). Whether the same conscious affective processes determine such ‘optimistic’ or ‘pessimistic’ biases in human and non-human animals remains to be established. For example, the finding that even some invertebrate species show judgement biases raises questions as to whether the phenomenon observed in animals involves any conscious component of affect. It may be that in some species valenced affective states, embodied in neural activity, drive changes in decision-making in the absence of any conscious experience, whilst in others a conscious component is involved as appears to be the case in humans (Mendl et al., 2011; Mendl and Paul, 2016).

## 8 Conclusions

The study of affective consciousness in animals falls squarely at the intersection of two longstanding controversies in psychological science – the relationship between consciousness and emotion and the measurement of nonhuman, and nonverbal, consciousness. Accordingly, the strands of empirical evidence and theoretical argument reviewed here are both richly diverse and hotly contested. But though it is beset by the twin enigmas of conceptualizing emotion and measuring consciousness, the study of animal affective consciousness is nonetheless of major potential importance, both for practical problems in animal welfare and for our efforts to get a clear view of our evolutionary kin, near and distant. We have adopted a componential view of emotions (reviewed in Section 3), in which conscious feelings constitute one component in a complex syndrome of related cognitive, motivational, expressive, and behavioural processes. And we have especially highlighted the implications of NCAC theories for a scientific understanding of how conscious feelings can, and cannot, empirically dissociate from other components of emotion, both within and across species.

In posing questions about conscious affect in animals, much (though not all^1^) work starts with the human case, where understanding is facilitated by subjects’ emotional reports (as well as the informal introspection the researcher employs in interpreting such reports). The human models are then used to identify candidate criteria for conscious emotion, which can be applied to observations of brain, behaviour, and physiology in different animal species. Research in this program can, in turn, be roughly divided into two classes – a *wide-focus* approach, which begins with general models of human consciousness (Section 4), and a *narrow-focus* approach, which sets out from specific models of human emotion (Section 5). The two approaches inform one another, because emotional consciousness is one form of consciousness, and together they can suggest principles for the identification of conscious affect in the absence of subjective report (Sections 6 and 7).

As our review illustrates, wide- and narrow-focus studies alike present a mixed picture of promising developments and enduring controversy. In our view, an especially promising strategy is to explicitly link proposed neurofunctional analyses of consciousness in general with a componential view of emotion in particular. This strategy is generative, suggesting novel potential resolutions to questions about conscious animal affect. Nonetheless, the stubborn persistence of core controversies (what kinds of cognition does consciousness require, and what kinds of emotional response require consciousness?) bars anything like a consensus choice among the candidate resolutions at present.

**Fig. 1:**
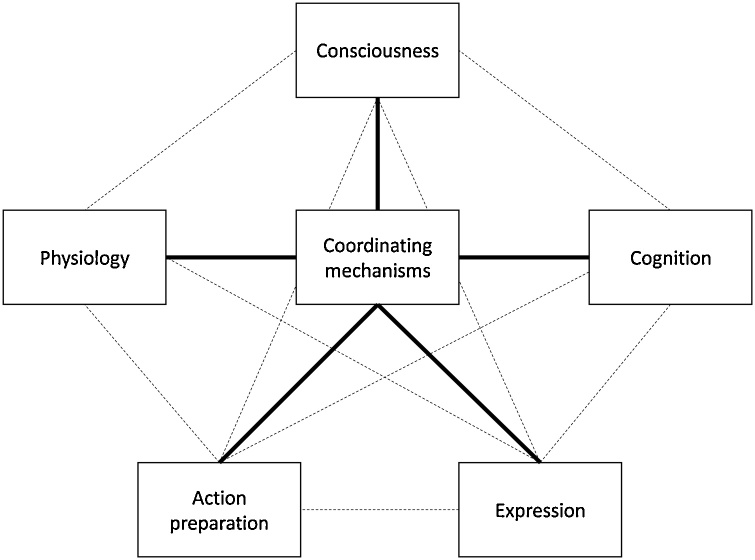
Componential framework for conceptualizing emotion. The five outer boxes depict component processes in emotion, similar to those identified in Scherer (2005a,b). The central box stands for possible central mechanisms (at cortical and/or subcortical levels) which may help to coordinate some or all of the components. Actions of the hypothetical central mechanisms are represented by solid lines, direct interactions between the five component processes by dashed lines.

As an example of this dynamic, consider Fig. 1 and its depiction of the componential view of emotion. Here, five components of emotion (Scherer, 2005a,b) are conceptually distinguished, and the task for emotion researchers is to explain their empirical coordination in emotional responses. Such explanations may refer to hypothesized “coordinating mechanisms” that coherently control the component mechanisms (the solid lines in Fig. 1) and/or to direct links between the component mechanisms themselves (dashed lines). It is important to emphasize, however, that Fig. 1 does not, on its own, constitute a model of emotion. Rather, it supplies a conceptual framework within which empirical questions about emotion can be posed – questions which an adequate model, drawing on both wide- and narrow-focus empirical approaches, must answer. Most importantly: (1) how is the coordination of the different components of an emotional response achieved? And (2) do the various components – including emotional consciousness – play comparable or unequal roles in the process of cross-component coordination?

**Fig. 2:**
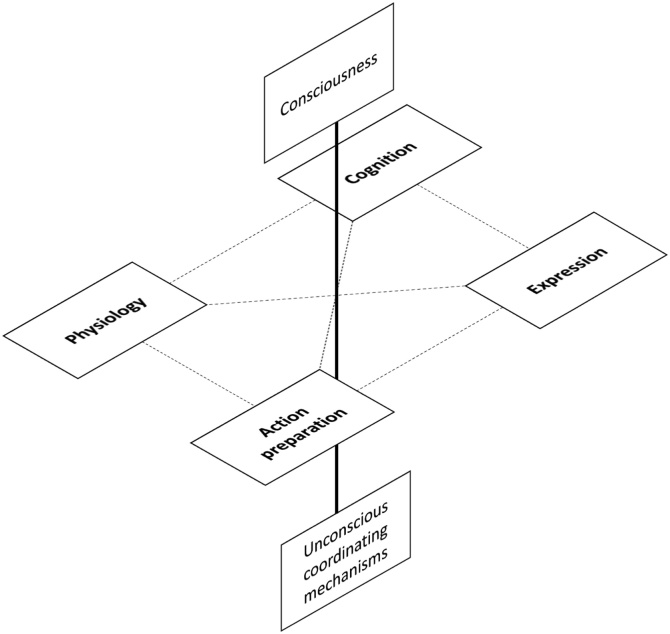
A possible neurofunctional interpretation of the componential framework. A GW model of conscious emotion is assumed for illustrative purposes. In this model, consciousness functions to globally integrate modular processors for the flexible control of cognition and action. On this view, consciousness is expected to play a central role in coordinating component processes, at least for those emotions which exhibit high levels of integration (i.e., responsiveness to a wide range of information inputs) and flexibility (i.e., adaptive sensitivity to a wide range of contexts). The model also allows for distinct unconscious coordinating mechanisms that may generate more stereotyped (aspects of) emotional responses.

Different models of emotion, drawing on different views of the functional role(s) of consciousness, suggest different answers to these two critical questions. As an illustration, Fig. 2 shows how one model of conscious emotion, derived from a subset of the research reviewed here, would resolve these questions. In this model, a GW perspective on affective consciousness is assumed. That is, consciousness – affective and otherwise – is assumed to be linked to thalamocortical broadcasting of selected information for the flexible coordination of cognition and action. If consciousness is inherently linked to this coordination function, it will presumably be essential for some aspects of the coordination of component processes in human emotion. Returning to Fig. 1, this GW-inspired viewpoint would then suggest that the “coordinating mechanisms” are not neatly separable from the “consciousness” component. Rather, the consciousness component constitutes part of the coordinating mechanisms (though further unconscious mechanisms, specific to emotion, may also play a role in coordinating an emotional response). Fig. 2 shows how this neurofunctional model of conscious emotion unpacks and relates the “flat” uninterpreted relations in Fig. 1. In this way, the model offers one possible answer to the critical questions of how the emotion components relate to the coordinating process and to one another (consciousness, unlike the other components, is part of a posited central coordinating mechanism). It suggests, in turn, criteria for affective consciousness in the absence of subjective report (i.e. does the affective response reflect a level of integration and flexibility that requires the operation of the GW?).^2^

The model in Fig. 2 illustrates how a neurofunctional analysis of consciousness can flesh out the componential framework for emotion, implying conditions under which consciousness can(not) dissociate from the other emotion components, and hence providing principled criteria whereby consciousness can be inferred from observation of the other components. To be sure, the neurofunctional analysis of conscious emotion (a GW view) assumed in Fig. 2 is not the only available one, and it is not definitively established by the evidence reviewed here. Alternative (e.g., HOT) neurofunctional analyses may assign the consciousness component in Fig. 1 a more peripheral functional role, implying readier dissociability from other components, and hence requiring more stringent criteria for the identification of conscious feelings. At the other end of the spectrum, some views associate basic forms of consciousness (or sentience) with more elementary nervous system functions, implying that consciousness accompanies even component-responses with minimal complexity or coordination.

Nonetheless, the example illustrates the logic of leading approaches to the study of conscious emotion, highlighting both their promise and their limitations. On the one hand, developing theories of the NCAC suggest substantive interpretations of the componential framework, from which principled criteria for affective consciousness in nonverbal creatures can be derived. On the other hand, the search for NCACs itself remains closely bound up with longstanding controversies in the conceptualization of both consciousness and emotion. It is inseparable from fundamental questions, still not adequately resolved, about when, how, and why conscious experiences can be inferred from behavioural responses when subjective report is unavailable. The merging of a componential view of emotion with a neurofunctional analysis of consciousness thus opens up promising new paths toward a scientific understanding of animal affective consciousness, but also shines a sobering light on the obstacles that lie in their way.

## Funding

The idea for this paper arose originally from discussions held between Michael Mendl, Piotr Winkielman and Marco Tamietto while attending Lorenz Centre workshop “Comparative affective science: The intersection of biology and psychology” (NIAS: Koninklijke Nederlandse Academie van Wetenschappen). It was not the product of any specific grant from funding agencies in the public, commercial or not-for-profit sectors. Elizabeth S. Paul is supported by BBSRC grants BB/P019218/1 and BB/T002654. Shlomi Sher is supported by a Scholar Award from the James S. McDonnell Foundation. Piotr Winkielman is supported by UCSD Academic Senate Grant (RS147R).

## Declaration of Competing Interest

None.

## Footnotes

1 For an instructive recent debate on the strengths and drawbacks of theories of emotion primarily derived from human versus animal studies, see Panskepp et al. (Panksepp et al., 2017).

2 Importantly, note that on this view, this question goes beyond the simple issue of the empirical correlation between component-responses – a correlation which empirical research has shown to be weak (Mauss et al., 2005). Instead, the question concerns the flexible, adaptive coordination of emotion components in response to specific contexts.
